# Reconstruction of *de novo* verazine biosynthesis in *Saccharomyces cerevisiae* and computational analysis of cholesterol-binding geometry for 22*R* hydroxylation by *Vc*CYP90B27 to dissect the cyclopamine biosynthetic pathway

**DOI:** 10.48130/els-0026-0008

**Published:** 2026-08-12

**Authors:** Chang Li, Quanwei Yu, Fang Yue, Xizi Wang, Yazhou Zhao, Jianfeng Zhang, Wei Song, Zheyong Xue

**Affiliations:** 1 Beijing Life Science Academy, Beijing 102209, China; 2 Department of Medicinal Chemistry and Natural Medicine Chemistry, College of Pharmacy, Harbin Medical University, Harbin 150081, China; 3 Heilongjiang Provincial Institute for Drug Control, Harbin 150088, China; 4 Key Laboratory of Saline-alkali Vegetation Ecology Restoration, Ministry of Education, College of Life Science, Northeast Forestry University, Harbin 150040, China; 5 State Key Laboratory of Rice Biology and Breeding, Northern Center of China National Rice Research Institute, China National Rice Research Institute, Hangzhou 310006, China

**Keywords:** *Saccharomyces cerevisiae* BY-SQ1, Cyclopamine, Verazine, Biosynthetic pathway, Steroidal alkaloid

## Abstract

Cyclopamine, a typical isosteroidal alkaloid identified as a hedgehog pathway inhibitor, serves as a crucial molecular scaffold for the semi-synthesis of drugs treating nevoid basal cell carcinoma. Verazine is a key intermediate in the biosynthetic pathway of cyclopamine. In this study, we reconstructed the biosynthetic pathway of verazine in engineered *Saccharomyces cerevisiae* BY-SQ1, obtaining a strain that produced verazine with a yield of 265.38 μg/L. Furthermore, during a 1,000-ns classical MD trajectory, cholesterol retained a broadly consistent heme-facing pose within *Vc*CYP90B27. The modeled pro-*R* C22 hydrogen remained closer to the heme Fe center on average than the pro-*S* hydrogen (4.01 vs 4.65 Å), revealing a persistent orientational bias in the bound ensemble. MM-GBSA and contact analyses further indicated that hydrophobic packing around the steroid scaffold and side chain may help maintain this asymmetric presentation. This research not only lays a foundation for the biosynthesis of cyclopamine and other steroidal alkaloids, but also provides a structural rationale for how cholesterol recognition by *Vc*CYP90B27 may contribute to the observed 22*R* hydroxylation and identifies candidate residues for experimental evaluation.

## Introduction

Isosteroidal alkaloids represent a distinctive class of alkaloids noted for their unique structural characteristics and significant pharmacological activities. These compounds possess a wide array of biological properties, including analgesic, antiasthmatic, antihypertensive, anti-inflammatory, and antitumor effects^[1−3]^. Typical isosteroidal alkaloids encompass the cevanine, veratramine, and jervine types, represented by compounds such as verticine, veratramine, and cyclopamine^[1,4−8]^. Despite their significant pharmacological potential, the practical application of these compounds is hampered by several inherent limitations. Their natural content in plant materials is generally low, and the source plants themselves often require extended growth cycles. Moreover, the processes of synthesis, separation, and purification are technically demanding, rendering the extraction process difficult to scale.

Cyclopamine, a typical isosteroidal alkaloid classified as a jervine-type steroidal alkaloid with a C-nor-D-homosteroidal skeleton, was first isolated from *Veratrum grandiflorum* in 1965^[9]^*.* Following this discovery, a new class of chemotherapeutics, hedgehog pathway inhibitors, has been developed and approved by the Food and Drug Administration (FDA) for the treatment of cancers, most notably basal cell carcinoma and acute myeloid leukemia^[10]^. Cyclopamine has also served as a molecular scaffold for the semi-synthetic derivative patidegib, which has received orphan drug approval for the treatment of nevoid basal cell carcinoma^[11]^. However, the yield of cyclopamine from plant sources is limited^[12]^. The total synthesis of cyclopamine is also time-consuming and environmentally burdensome, achieving an overall yield of only 1.4% over a 16-step longest linear sequence^[13]^. A promising alternative for scalable production is biosynthesis using engineered plants or microorganisms. Verazine is a key intermediate in the biosynthesis of cyclopamine, and itself exhibits various bioactivities, including anti-inflammatory and antifungal properties^[14,15]^. It is biosynthesized from cholesterol via a five-step pathway^[16]^. Several strategies have been successfully employed to produce verazine at small scales, including semi-synthesis from diosgenin, as well as biosynthesis via heterologous expression in *Spodoptera frugiperda* and in *Camelina sativa* seeds^[17−19]^. Our previous report described the pathway construction in *Nicotiana benthamiana*, the combination of *Vn*CYP90B27 (from *Veratrum nigrum*), *Vn*CYP94N2, *Vc*GABAT (from *V. californicum*), and *Vc*CYP90G1, producing the highest verazine yield of 0.53 mg/g dry weight (DW)^[20]^. Meanwhile, Winegar et al. established heterologous biosynthesis of verazine in *Saccharomyces cerevisiae*, achieving a yield of 83 μg/L^[16]^. Hong et al. reported the fermentation yield in yeast with 175 µg/L^[21]^.

Previously, we engineered *S. cerevisiae* to generate strain BY-SQ1^[22,23],^ which achieved improved squalene production of 1.24 g/L. In this study, we reported the construction of the biosynthetic pathway for verazine using this engineered yeast BY-SQ1 as a heterologous host. In addition, *Vc*CYP90B27 is a key enzyme in this pathway and catalyzes the 22*R*-hydroxylation of cholesterol. Within its chiral heme pocket, the binding geometry of cholesterol shapes how the two stereotopically distinct C22 hydrogens are presented to the catalytic center and may therefore contribute to the stereochemical outcome.

## Materials and methods

### Source of chemicals and DNA

Genes *VcCYP90B27*, *VcCYP94N1*, *VcCYP90G1*, *VcGABAT*, *DrDHCR7*, *ERG1*, *GgDHCR24*, and *VvCPR* were optimized and synthesized by Integrated DNA Technologies. Primers for PCR amplification were purchased from Integrated DNA Technologies. pEASY®-Blunt Simple Cloning Kit was purchased from TransGen Biotech Co., Ltd.

### Plasmid construction and verification

All plasmids used in this study were constructed according to the following protocol. Genes, promoters (e.g., pTEF1, pFBA1, pFBA1, and pPGK1), and terminators (e.g., ADH1t, TDH2t, FBA1t, and CYC1t) were amplified by polymerase chain reaction (PCR) on a Gene Explorer Thermal Cycler (Bioer). PCR was performed with the Phanta Max Super-Fidelity DNA polymerase (New England Biolabs, M0493S), Phanta^®^Max Buffer (Vazyme), and dNTP mix (Vazyme). PCR products and the DNA Marker (Takara) were mixed with 10 × gel loading dye and loaded into 1% (w/v) agarose gels stained with Super Red nucleic acid stain (Biosharp). The agarose gel was run in an Elite 300 Plus Electrophoresis System (Wealtec) at 120 V for 25 min. Gels were imaged on a Dolphin-Doc Imager (Wealtec). The target DNA was extracted from the gel bands using the E.Z.N.A.® Gel Extraction Kit (Omega Bio-Tek). Then, pEASY®-Blunt Simple plasmids were constructed from DNA amplicons. Subsequently, the plasmids were transformed into *Escherichia coli* (*E. coli*) DH5α. Individual *E. coli* colonies were selected from the plates and used to inoculate 5 mL of LB medium containing 100 μg/mL kanamycin. These liquid cell cultures were incubated at 37 °C overnight with shaking at 200 rpm. Next, plasmids were extracted from liquid cell cultures using the Plasmid Mini Kit I D6943 (Omega Bio-tek). Successful plasmid construction was confirmed by whole plasmid next-generation sequencing. All primers, genes, strains used were listed in Supplementary Tables S1−S3.

### *S. cerevisiae* strain construction and verification

All *S. cerevisiae* strains used in this study were constructed according to the following protocol and stored in our laboratory. For transformations involving the deletion of native genes and their replacement with heterologous genes—specifically, pFBA1-ERG1-CPS1t, pTEF1-*Dr*DHCR7-PDC1t, pTDH3-*Gg*DHCR24-ENO2t, PTEF1-*Vv*CPR-TCPS1, pTEF1-*Vc*CYP90B27-ADH1t, pFBA1-*Vc*CYP94N1-TDH2t, pTDH3-*Vc*tGABAT-FBA1t, and pPGK1-*Vc*CYP90G1-CYC1t—positive transformants were selected using Synthetic Defined (SD) agar media lacking the appropriate auxotrophic components (uracil, histidine, leucine, or tryptophan)^[24]^. Yeast strains were routinely cultivated at 30 °C in standard Yeast Peptone Dextrose (YPD) medium (1% w/v yeast extract, 2% w/v peptone, 2% w/v glucose) or SD medium (0.67% w/v yeast nitrogen base without amino acids, 2% w/v glucose).

### Liquid cell culture of *S. cerevisiae* for GC-MS and LC-MS characterization

For metabolite production, liquid cell cultures were grown on a 20 mL scale. Glycerol stocks of yeast strains were streaked onto 1 × YPD plates and incubated at 28 °C for 1–2 d. A single, well-isolated colony was selected to inoculate 20 mL of 1 × YPD medium. The liquid culture was incubated at 28 °C for 4 d with shaking at 200 rpm. Cells (2 mL) were then pelleted by centrifugation (10,000 rpm, 1 min), and the supernatant was discarded. The cell pellet was resuspended in 1 mL of saponification solution (10% KOH dissolved in 80% anhydrous ethanol) containing 10 μg/mL 5α-cholestane. After incubation at 70 °C for 2–4 h, the mixture was allowed to dry overnight with the lid open. Subsequently, 500 μL of chromatographic-grade ethyl acetate was added, and the mixture was suspended by sonication, followed by the addition of 500 μL of ultrapure water for extraction. After centrifugation at 12,000 rpm for 5 min, the ethyl acetate layer was carefully transferred to a new centrifuge tube. An aliquot of 50 μL of the supernatant was pipetted into a sample vial, dried by spinning for 15 min, and then mixed with 30 μL of the derivatization reagent. The mixture was allowed to stand at 70 °C for 1 h, and finally diluted with 70 μL of chromatographic-grade ethyl acetate prior to instrument analysis. Samples were then analyzed on a GC-MS system (Thermo Scientific™ TRACE™ 1310 with Thermo Scientific™ ISQ™ 7000) equipped with a ZB-5 HT column (0.25 mm × 30 m, Thermo Fisher Scientific), using a previously reported heating program^[25]^.

Samples were analyzed by HPLC coupled with a Triple TOF 6600 MS (AB/SCIEX, USA) operated under positive electrospray ionization mode with a Phenomenex Kinetex C18 column (150 mm × 4.6 mm, particle size 2.6 μm, pore size 100 Å). Mobile phase A was aqueous water with 0.1% formic acid, and B was acetonitrile containing 0.1% formic acid. The flow rate was maintained at 0.4 mL/min. The gradient elution procedure was set as follows: 0–2 min, constant 5% B; 2–10 min, linear gradient from 5% to 100% B; 10–12 min, isocratic elution with 100% B; 12–13 min, from 100% down to 5% B; 13–15 min, 5% B.

### Statistical analysis

Each strain was evaluated in three independent replicates (*n* = 3). Differences between groups were analyzed for significance using either a t-test or one-way ANOVA.

### MD simulations

To construct a model of the *Vc*CYP90B27-heme-cholesterol bound complex, the crystal structure of *Arabidopsis* CYP90B1 (PDB 6A15) was used as a template. After AlphaFold prediction and structural alignment, the heme cofactor and cholesterol substrate from 6A15 were placed in the *Vc*CYP90B27 pocket, followed by energy minimization. After NVT and NPT equilibration, a single unbiased 1,000-ns all-atom classical MD production trajectory was generated in GROMACS using the AMBER14SB force field. RMSD values for the protein backbone, binding site, and cholesterol, together with per-residue RMSF values and conformational overlays, were used to characterize structural drift, local flexibility, and retention of the bound pose. The two stereotopically distinct C22 hydrogens were designated pro-*R* and pro-*S* in the model, and their Fe-to-H distances were analyzed as time series and probability-density distributions to compare their relative presentation toward the heme throughout the trajectory. MM-GBSA provided a model-dependent end-point estimate of binding energetics for trajectory snapshots, while per-residue decomposition and non-covalent contact analysis were used to identify interactions and candidate residues that may help maintain the observed binding geometry (detailed methods are provided in the Supplementary Text 1).

## Results and discussion

The yeast strain BY-SQ1 was previously engineered from OCP1, an isolated variant derived from CEN. PK2-1C that harbored an orthogonal cytosolic FPP biosynthetic pathway comprising the genes *ERG10*, *ERG13*, *HMG1*/*HMG2*, *ERG12*, *ERG8*, *ERG19*, *IDI1*, and *ERG20*. BY-SQ1 was selected as the starting point for this work due to its previously engineered capacity to produce high levels of squalene (1.24 g/L).

**Figure 1:**
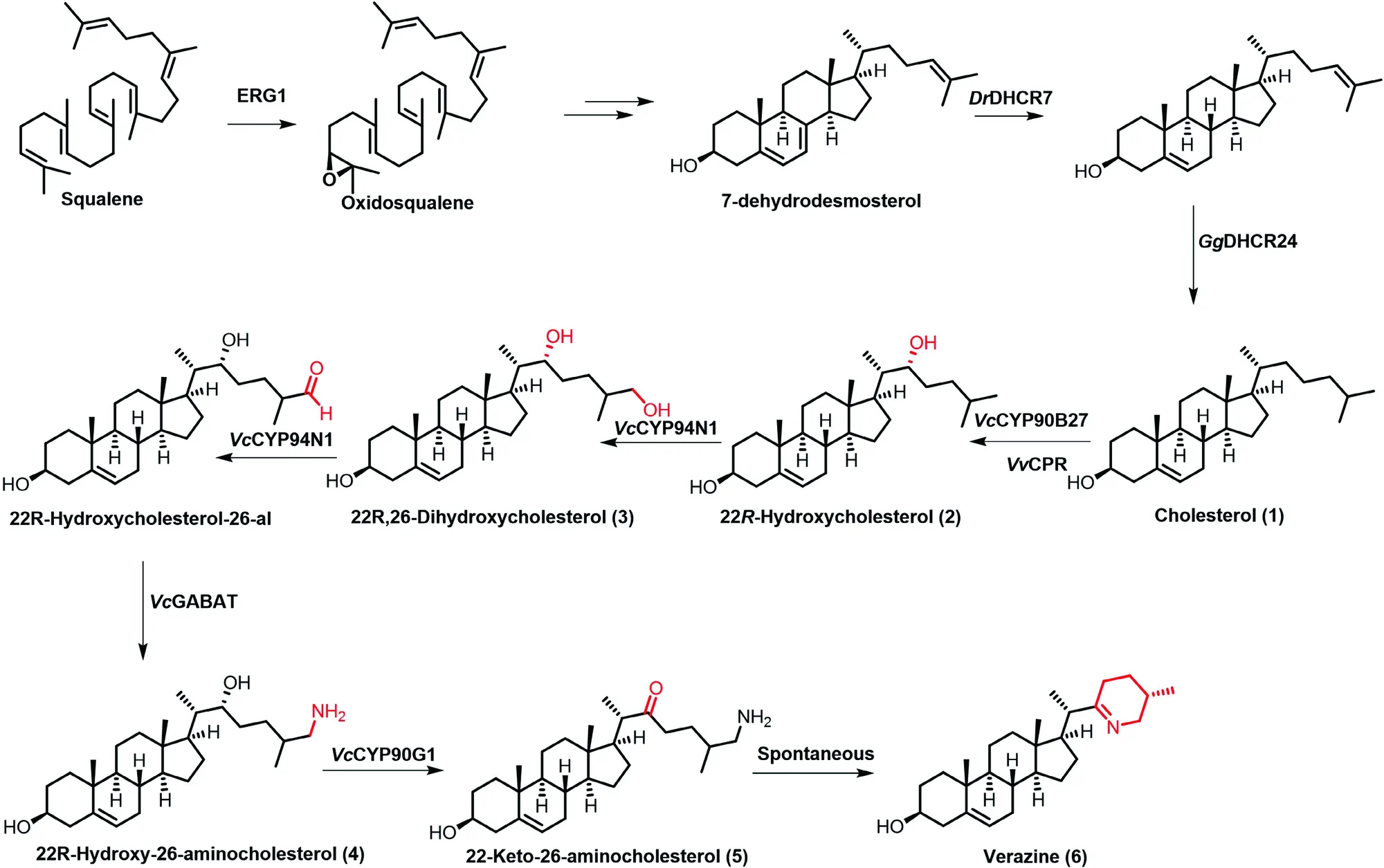
BY-SQ1 was engineered to biosynthesize verazine (6) from squalene through inducible expression of an engineered biosynthetic pathway. (Dr: *Danio rerio*; Gg: *Gallus gallus*; Vc: *Veratrum californicum;* Vv*: Vitis vinifera*).

Because 7-dehydrodesmosterol can be converted to cholesterol (**1**) by DHCR7 (Δ7[8]-reductase) and DHCR24 (Δ24[25]-reductase)^[16]^, the genes encoding the enzyme SE (squalene epoxidase, *ERG1*) as well as *Dr*DHCR7 and *Gg*DHCR24 from *Danio rerio* and *Gallus gallus*, respectively, were integrated into strain BY-SQ1 (Fig. 1). This modification resulted in high-level cholesterol production, and the resulting strain was named CEN-Chol^[16]^. Engineering CEN-Chol through heterologous expression of *VvCPR* led to a modest increase in cholesterol production, and the resulting strain was designated CEN-CPR.

**Figure 2:**
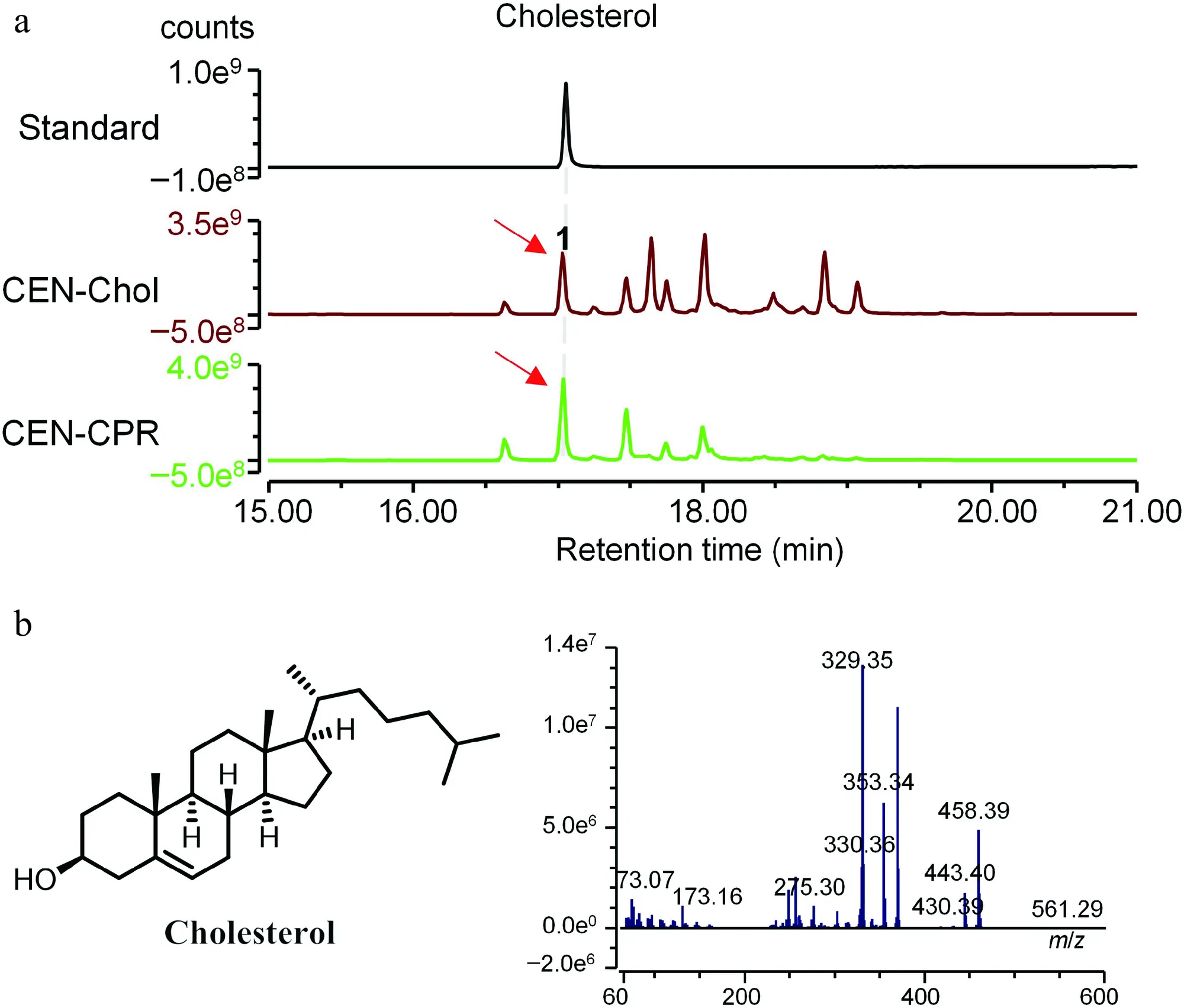
The characterization of cholesterol (1) in CEN-Chol and CEN-CPR strains by GC-MS. (a) TIC spectrum of each strain compared to the standard sample; (b) the structure of cholesterol (1) and its MS spectrum.

These strains were incubated in SD-Trp-Leu medium with shaking for 24 h during the growth phase and for an additional 72 h in YPD medium during the production phase. Cholesterol (**1**) produced in each strain was confirmed by matching gas chromatography-mass spectrometry (GC-MS) retention times and mass spectra with those of a commercial standard (Fig. 2). Cholesterol production in strains CEN-Chol and CEN-CPR reached 79.92 mg/L and 115.37 mg/L, respectively.

The first step in the conversion of cholesterol (1) to verazine (6) was the cytochrome P450 (CYP450)-mediated C22 hydroxylation of cholesterol. *Vc*CYP90B27 from *V. californicum* was selected as the starting point for constructing a 22*R*-hydroxycholesterol-producing strain.

**Figure 3:**
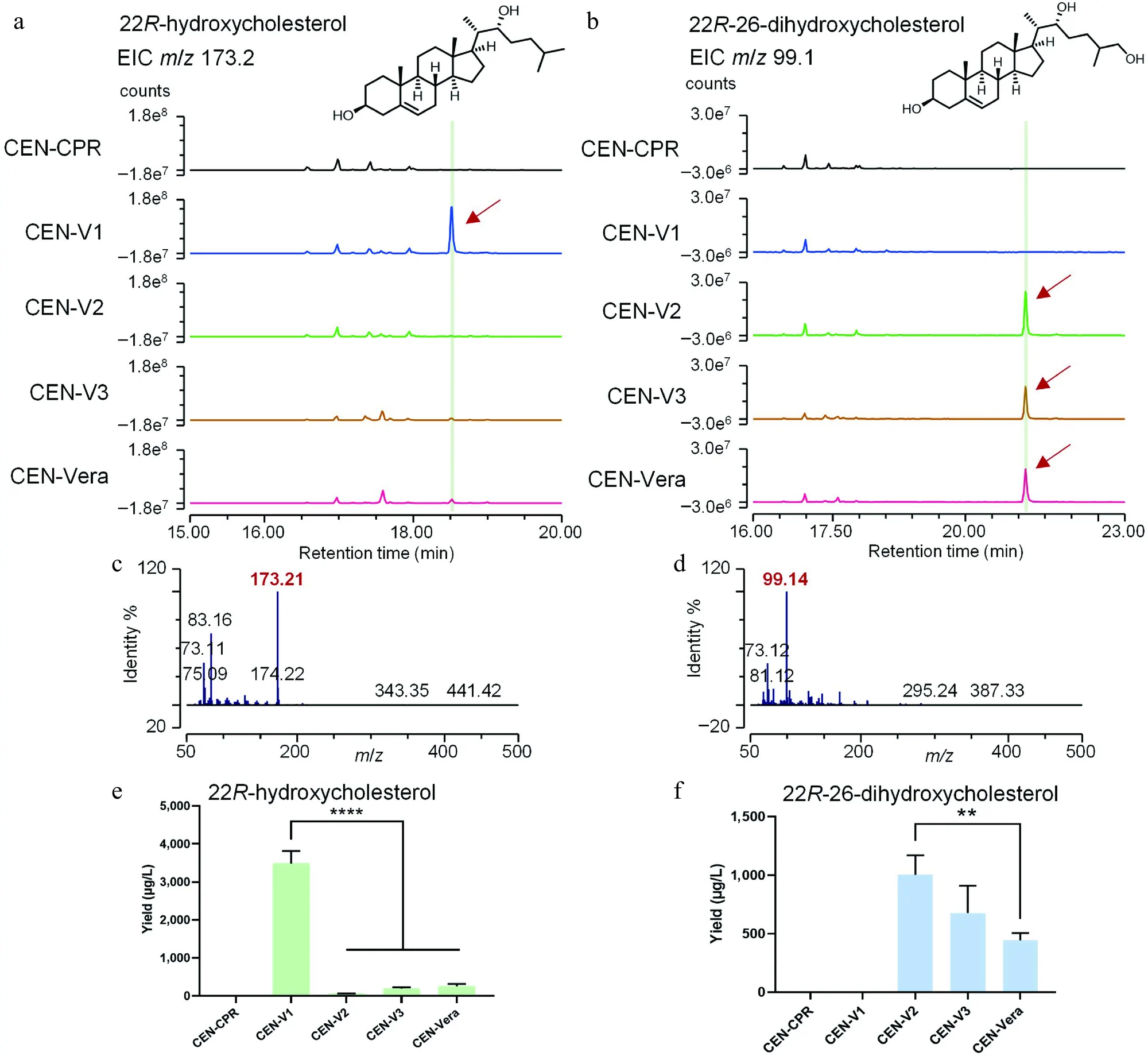
The characterization of 22*R*-hydroxycholesterol (2) as well as 22*R*-26-dihydroxycholesterol (3) in different strains by GC-MS. (a), (b) EIC spectra of the four strains of CEN-V1, CEN-V2, CEN-V3, and CEN-Vera at *m*/*z* 173 and 99, respectively, and CEN-CPR as a control. (c), (d) MS spectra of 22*R*-hydroxycholesterol (2) and 22*R*-26-dihydroxycholesterol (3). (e), (f) Yields of 22*R*-hydroxycholesterol (2) and 22*R*-26-dihydroxycholesterol (3) in different strains. (** *p* < 0.01, **** *p* < 0.0001).

**Figure 4:**
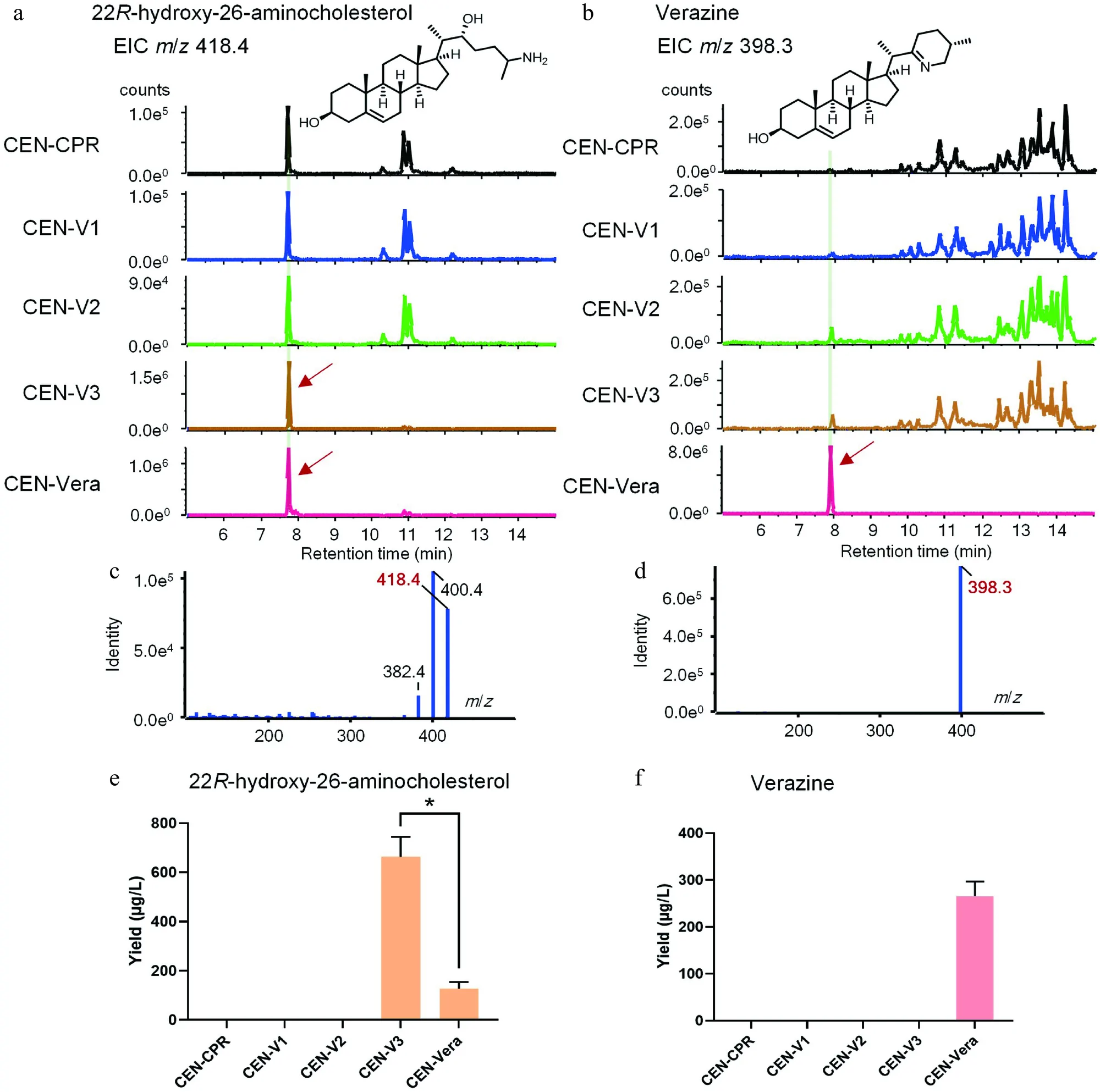
The characterization of 22*R*-hydroxy-26-aminocholesterol (4) as well as verazine (6) in different strains by LC-MS in positive mode. (a), (b) EIC spectra of the four strains of CEN-V1, CEN-V2, CEN-V3, and CEN-Vera at *m*/*z* 418 and 398, respectively, and CEN-CPR as a control. (c), (d) MS spectra of 22-hydroxy-26-aminocholesterol (4) as well as verazine (6). (e), (f) Yields of 22-hydroxy-26-aminocholesterol (4) as well as verazine (6) in the different strains. (* *p* < 0.05).

This enzyme has also been used in *Spodoptera frugiperda* Sf9 insect cell culture and in *Camelina sativa* seeds^[16]^. The gene encoding *Vc*CYP90B27 was integrated into the ATF2 locus of strain CEN-CPR. The resulting strain, CEN-V1, was incubated with shaking for 4 d, yielding 22*R*-hydroxycholesterol of 3,482.83 μg/L (Fig. 3). The second step in this pathway was the CYP450-mediated C26 oxidation of 22*R*-hydroxycholesterol (2). The genes encoding *Vc*CYP90B27 and *Vc*CYP94N1 were co-integrated into strain CEN-CPR, yielding strain CEN-V2. This strain produced 22*R*-26-dihydroxycholesterol (3) to 1,003.05 μg/L (Supplementary Table S4). The third step in this pathway was the γ-aminobutyric acid (GABA) aminotransferase-mediated C26 transamination of 22-hydroxycholesterol-26-al. The genes encoding *Vc*CYP90B27, *Vc*CYP94N1, and *Vc*GABAT were co-integrated into strain CEN-CPR, yielding strain CEN-V3. This strain produced 22-hydroxy-26-aminocholesterol (4), as confirmed by LC-MS analysis, with a yield of 662.98 μg/L (Fig. 4). The fourth and fifth steps in this pathway were the CYP450-mediated C22 oxidation of 22-hydroxy-26-aminocholesterol (4), followed by spontaneous cyclization via imine formation of 22-keto-26-aminocholesterol (5). The genes encoding *Vc*CYP90B27, *Vc*CYP94N1, *Vc*GABAT, and *Vc*CYP90G1 were integrated into strain CEN-CPR, yielding strain CEN-Vera. Although 22-keto-26-aminocholesterol (5) could not be directly detected, this strain spontaneously produced verazine (6) at 265.38 μg/L.

Before C–H activation could occur, cholesterol must adopt a heme-facing orientation within *Vc*CYP90B27 (Supplementary Table S5), a key enzyme in this pathway that catalyzes the 22*R*-hydroxylation of cholesterol. The extent to which the active site enforces an asymmetric presentation of the two C22 hydrogens was therefore critical to the observed 22*R* selectivity. To probe this, classical MD simulations were employed, providing a time-resolved description of the bound conformational ensemble through RMSD, RMSF, conformational overlays, and interatomic distance analyses^[26,27]^.

**Figure 5:**
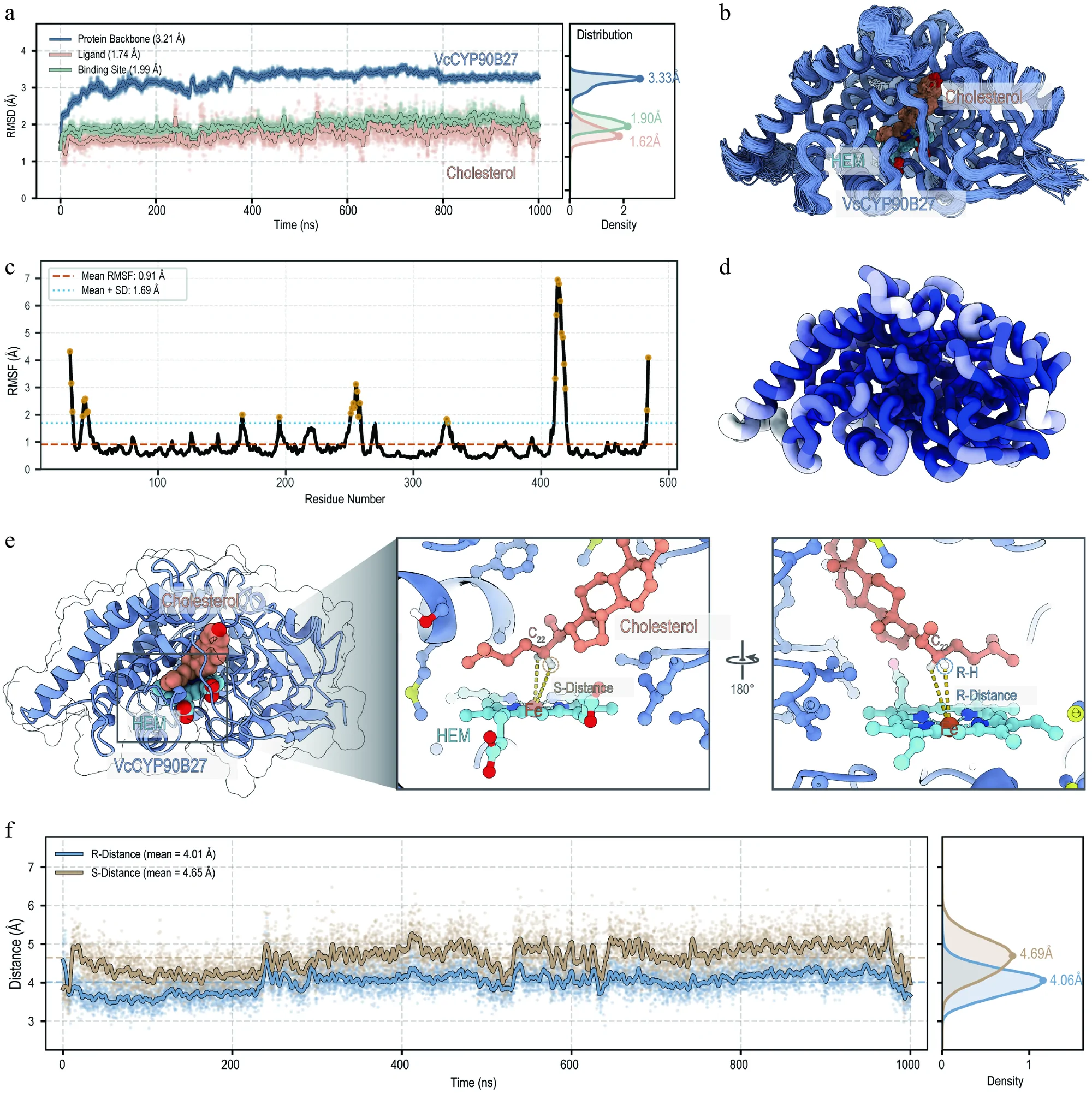
Conformational behavior and trajectory-based sampling of heme-proximal cholesterol-binding geometry in the *Vc*CYP90B27-heme-cholesterol complex during a single 1,000-ns classical MD trajectory. (a) RMSD trajectories and probability-density distributions for the protein backbone (3.21 Å), binding site (1.99 Å), and cholesterol (1.74 Å). (b) Overlay of representative conformations from the later portion of the trajectory. (c) Per-residue RMSF profile, with an overall mean of 0.91 Å. (d) A three-dimensional surface representation colored by RMSF. (e) Relative positions of the two stereotopically distinct C22 hydrogens, designated pro-*R* and pro-*S* in the model, and the heme iron (Fe). (f) Trajectories and probability-density distributions of the Fe-to-pro-*R* H and Fe-to-pro-*S* H distances over 1,000 ns.

After the initial structural adjustment, the binding-site and cholesterol RMSD traces fluctuated within comparatively narrow ranges (Fig. 5a, b). Their mean RMSD values (1.99 and 1.74 Å, respectively) were lower than that of the protein backbone (3.21 Å), and the conformational overlay showed that cholesterol retained a broadly similar orientation in the pocket. The mean protein RMSF was 0.91 Å, with higher mobility concentrated in several localized regions (Fig. 5c, d). No large-scale flipping of cholesterol was observed. Together, these features indicate that the local binding geometry was maintained over the sampled trajectory and provide a consistent basis for comparing the two C22 hydrogens.

Against this maintained binding geometry, the two Fe-to-H distance traces varied in parallel over much of the trajectory but retained a clear average offset (Fig. 5e, f). The hydrogen designated pro-R in the model remained closer to the heme Fe on average than the pro-S hydrogen (4.01 vs 4.65 Å), corresponding to a mean difference of 0.64 Å. This time-resolved separation indicates that the C22 methylene was presented asymmetrically across the sampled bound ensemble rather than only in a few isolated snapshots.

The corresponding probability-density distributions partially overlapped as the complex fluctuated, but the pro-R distribution was shifted toward shorter Fe-to-H distances relative to the pro-S distribution. The combined time-series and distribution behavior therefore described an orientational bias rather than an exclusive separation between two geometries. In the chiral active-site environment, the direction of this bias was consistent with the experimentally observed 22*R* product and provided a structural rationale for how substrate positioning may contribute to the stereochemical outcome of *Vc*CYP90B27.

The persistence of the cholesterol pose was accompanied by a network of non-covalent contacts within the binding cavity. MM-GBSA decomposition for trajectory snapshots (Supplementary Fig. S1) showed favorable van der Waals and lipophilic contributions and an unfavorable polar solvation contribution, consistent with predominantly nonpolar complementarity between cholesterol and the pocket. Per-residue decomposition highlighted ILE112, MET116, MET213, and PRO467, while contact analysis identified frequent interactions involving ALA287, ILE112, and PHE359 around the steroid scaffold and side chain. Together, these observations were consistent with multipoint hydrophobic packing that may limit large-scale cholesterol reorientation and thereby help maintain the heme-facing pose and the asymmetric C22 presentation. These residues therefore represented candidate contributors to substrate positioning and provided focused sites for experimental evaluation.

Because only a single classical MD trajectory was analyzed, the RMSD, RMSF, distance probability-density distributions, and MM-GBSA estimates lack independent replication and between-trajectory uncertainty. In addition, no QM/MM reaction-profile calculation was performed in the present study. A rigorous reaction-level analysis remains a priority for future work, together with independent replicate simulations, convergence and uncertainty analyses, and targeted mutagenesis and kinetic measurements to test the proposed binding-orientation model and the roles of the candidate residues.

## Conclusions

In conclusion, although heterologous production of verazine in yeast has been reported previously, the highest reported yield was only 175 μg/L^[21]^. In this study, we systematically engineered the host strain from BY-SQ1 to CEN-Vera and successfully reconstructed the verazine biosynthetic pathway, achieving an increased production level of 265.38 μg/L. As a key biosynthetic intermediate of cyclopamine, improved verazine availability is expected to facilitate downstream pathway characterization and the identification of additional pathway intermediates and products^[14,28]^.

Complementing the pathway reconstruction, the 1,000-ns MD trajectory revealed that VcCYP90B27 maintains cholesterol in a broadly consistent heme-facing orientation, stabilized by a multipoint hydrophobic contact network. Within this bound ensemble, the pro-*R* hydrogen in the model remained, on average, closer to the heme iron than the pro-*S* hydrogen (4.01 vs 4.65 Å), producing a persistent orientational bias even though the distance distributions partially overlapped. Together, the observed pose retention, asymmetric C22 presentation, and the MM-GBSA and contact analyses furnish a coherent structural rationale for how substrate recognition may underpin the selective 22*R* hydroxylation. From this model, ILE112, MET116, MET213, PRO467, ALA287, and PHE359 emerged as candidate residues for substrate positioning, offering focused targets for experimental validation.

## SUPPLEMENTARY DATA

Supplementary data to this article can be found online.

## Data Availability

All data generated or analyzed during this study are included in this published article and its supplementary information files.
