# Supplementary material for: Reconstruction of de novo verazine biosynthesis in Saccharomyces cerevisiae and computational analysis of cholesterol-binding geometry for 22R hydroxylation by VcCYP90B27 to dissect the cyclopamine biosynthetic pathway
Source: Eng Life Sci. 2026 Aug 12;26(8):e008. doi: 10.48130/els-0026-0008 (PMC13599646; doi:10.48130/els-0026-0008)
Supplement: Supplementary file 1 [file els-2026-26-8-0008-S1.zip › 10.48130_els-0026-0008-Suppl-TableS1.pdf]

Table S1 Primers used in the study

Name	Primers
pTEF1-VvCPR-F	AAGTTTTAATTACAAACTCGAGATGCAATCATCCTCCGTAAAG
CPS1t-VvCPR-R	TTCAATCATTGCGCGGCGCGCCTATTACCAGACATCTCTCAAGTA
pTEF1-VcCYP90B27-F	CTAAGTTTTAATTACAAACTCGAGATGGCTATGGAATTGTTGTTGT
ADH1t-VcCYP90B27-R	CATAAGAAATTCGCGGCGCGCCTATTAGTCACCCAATGGTCTAAC
pFBA1-VcCYP94N1-F	AAGTAATACATATTCAAACTCGAGATGGATTTGCCATCTGCTTCAG
TDH2t-VcCYP94N1-R	AGGAGTTAAATTTAGGCGCGCCTATTAACAACCACGCTTTCTTTC
pTDH3-VcGABAT-F	CACACATAAACAAACAAACTCGAGATGGGATCCACTGAGGCGC
FBA1t-VcGABAT-R	TAATTTGAATTAACGGCGCGCCTATTAAGTTGCGGTATTCTGAGA
pPGK1-VcCYP90G1-F	TACAACAAATATAAAACACTCGAGATGACTCCATTAGTTGTTCTCT
CYC1t-VcCYP90G1-R	CGGTTAGAGCGGATGGCGCGCCTATTATTGGAGGAGCGAAAGCC
ATF2up-VcCYP90B27-F	CCACAATCTCAGGCTACATTGAACTCTGTAGGCCACCGATAAATATTGCGAAGTGATCCCCCACACACCAT
VcCYP94N1-VcCYP90B27-R	GGCTGGTATTGTTGTTCAAGCCAGCGGTGCGAGCGACCTCATGCTATACC
VcCYP90B27-VcCYP94N1-F	GCTTTCTCAGGTATAGCATGAGGTCGCTCGCACCGCTGGCTTGAACAACA
VcGABAT-VcCYP94N1-R	TTGTTGACGCTAACATTCAACGCTAGTATGCGAAAAGCCAATTAGTGTGA
VcCYP94N1-VcGABAT-F	CTTAGTATCACACTAATTGGCTTTTCGCATACTAGCGTTGAATGTTAGCG
VcCYP90G1-VcGABAT-R	TAAAAGCAATTACAGTATGGGAAGTCAATGTTGGAGGATAAAGTAAGCTA
VcGABAT-VcCYP90G1-F	CATAGTAGCTTACTTTATCCTCCAACATTGACTTCCCATACTGTAATTGC
ATF2dn-VcCYP90G1-F	AATAGATTAAATACAAAAGATTTATTATACATGCGAGGTAAGACTATTGTCTTCGAGCGTCCCAAAACCT
pTEF1-DrDHCR7-F	AAGTTTTAATTACAAACTCGAGATGATGGCTTCTGATAGAGTTA
PDC1t-DrDHCR7-R	AGAGATTAAATCGCGGCGCGCCTATTAAAAAATATTTGGCAACAATCT
pTDH3-GgDHCR24-F	CACATAAACAAACAAACTCGAGATGTCTGCTGTTTGGTCTTTG
ENO2t-GgDHCR24-R	TAGTTAAAAGCACTGGCGCGCCTATTAATGTCTAGCAGCTTTACAAA
ERG1-F	AAGTTTAATAACTCGAAAATTCTGCGTTATACTAGCGTTGAATGTTAGCG
ERG1-R	ATCTTGCAGTCCATCCTCGTCGCTTTATTATCGATAATATAGGTATACAAGAGCGACCTCATGCTATACC
δDNAup-F	TAGTATATTATCATATACGGTGTT
DrDHCR7-ERG1-R	TGAAGCTATGGTGTGTGGGGGATCACTTTTGACACTTGATTTGACACTTC
ERG1-DrDHCR7-F	AAAAAAAAGAAGTGTCAAATCAAGTGTCAAAAGTGATCCCCCACACACCA
GgDHCR24-DrDHCR7-R	GTTGACGCTAACATTCAACGCTAGTATTGGCAGTTTTGAATTGAGTAACC
DrDHCR7-GgDHCR24-F	ATAATGGTTACTCAATTCAAAACTGCCAATACTAGCGTTGAATGTTAGCG
Leu-GgDHCR24-R	CCTGAGTATTCCCACAGTTGTCGACATTTTTCAAACTGCAAATTCAAGAA
GgDHCR24-Leu-F	TCTTGAATTTGCAGTTTGAAAAATGTCGACAACTGTGGGAATACTCAGGT
δDNAdn-R	GCAGGTGTTGTTGTCTGTTG
