# Supplementary material for: Reconstruction of de novo verazine biosynthesis in Saccharomyces cerevisiae and computational analysis of cholesterol-binding geometry for 22R hydroxylation by VcCYP90B27 to dissect the cyclopamine biosynthetic pathway
Source: Eng Life Sci. 2026 Aug 12;26(8):e008. doi: 10.48130/els-0026-0008 (PMC13599646; doi:10.48130/els-0026-0008)
Supplement: Supplementary file 1 [file els-2026-26-8-0008-S1.zip › 10.48130_els-0026-0008-Suppl-TableS2.pdf]

**Table S2** Genes used for heterologous expression in this study

| Protein | Origin Species | Abbreviated protein name | GenBank Accession Code |
|---|---|---|---|
| Squalene epoxidase | *Saccharomyces cerevisiae* | ERG1 | NP_011691.1 |
| Δ7(8)-sterol reductase | *Danio rerio* | *Dr*DHCR7 | NP_958487.2 |
| Δ24(25)-sterol reductase | *Gallus gallus* | *Gg*DHCR24 | NP_001026459.1 |
| Cytochrome P450 reductase | *Vitis vinifera* | *Vv*CPR | According the the reference: Met Eng. 2022,70,115-128 |
| Cholesterol 22-hydroxylase | *Veratrum californicum* | *Vc*CYP90B27 | AJT59558.1 |
| 22-Hydroxycholesterol 26-oxidase | *Veratrum californicum* | *Vc*CYP94N1 | AJT59561.1 |
| 22-Hydroxycholesterol-26-al 26-transaminase | *Veratrum californicum* | *Vc*GABAT1 | AJT59569.1 |
| 22-Hydroxy-26-aminocholesterol 22-oxidase | *Veratrum californicum* | *Vc*CYP90G1 | AJT59566.1 |
