# Supplementary material for: Reconstruction of de novo verazine biosynthesis in Saccharomyces cerevisiae and computational analysis of cholesterol-binding geometry for 22R hydroxylation by VcCYP90B27 to dissect the cyclopamine biosynthetic pathway
Source: Eng Life Sci. 2026 Aug 12;26(8):e008. doi: 10.48130/els-0026-0008 (PMC13599646; doi:10.48130/els-0026-0008)
Supplement: Supplementary file 1 [file els-2026-26-8-0008-S1.zip › 10.48130_els-0026-0008-Suppl-TableS3.pdf]

Table S3 Strains with different genes constructed in the study

Name	Description
CEN-Chol	BY-SQ1:<br>*δDNA::*(P_{FBA1}-*ERG1*-T_{CPS1})-(P_{TEF1}-*DrDHCR7*-T_{PDC1})-(P_{TDH3}-*GgDHCR24*-T_{ENO2})-(P_{LEU}-*LEU*-T_{LEU})
CEN-CPR	CEN-Chol:<br>*ΔGAL80::*(*PTEF1-VvCPR-TCPS1*)-(P_{URA}-*URA*-T_{URA})
CEN-V1	CEN-CPR:<br>*ΔATF2::*(P_{TEF1}-*VcCYP90B27*-T_{ADH1})-(P_{HIS}-*HIS*-T_{HIS})
CEN-V2	CEN-CPR:<br>*ΔATF2::*(P_{TEF1}-*VcCYP90B27*-T_{ADH1})-(P_{FBA1}-*VcVYP94N1*-T_{TDH2})-(P_{HIS}-*HIS*-T_{HIS})
CEN-V3	CEN-CPR:<br>*ΔATF2::*(P_{TEF1}-*VcCYP90B27*-T_{ADH1})-(P_{FBA1}-*VcVYP94N1*-T_{TDH2})<br>-(P_{TDH3}-*VcGABAT*-T_{FBA1})-(P_{HIS}-*HIS*-T_{HIS})
CEN-Vera	CEN-CPR:<br>*ΔATF2::*(P_{TEF1}-*VcCYP90B27*-T_{ADH1})-(P_{FBA1}-*VcVYP94N1*-T_{TDH2})<br>-(P_{TDH3}-*VcGABAT*-T_{FBA1})-(P_{PGK1}-*VcCYP90G1*-T_{CYC1})-(P_{HIS}-*HIS*-T_{HIS})
