# Supplementary material for: Reconstruction of de novo verazine biosynthesis in Saccharomyces cerevisiae and computational analysis of cholesterol-binding geometry for 22R hydroxylation by VcCYP90B27 to dissect the cyclopamine biosynthetic pathway
Source: Eng Life Sci. 2026 Aug 12;26(8):e008. doi: 10.48130/els-0026-0008 (PMC13599646; doi:10.48130/els-0026-0008)
Supplement: Supplementary file 1 [file els-2026-26-8-0008-S1.zip › 10.48130_els-0026-0008-Suppl-TableS4.pdf]

Table S4 MS spectra of each compound in references

Name	MS spectrum in references	MS spectrum in this study
22*R*-hydroxycholesterol[1-2]	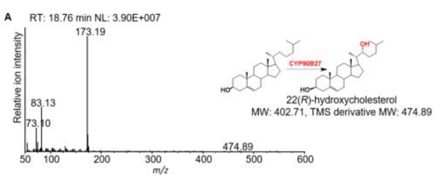	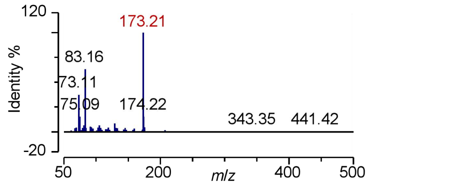
22*R*-26-dihydroxycholesterol[1]	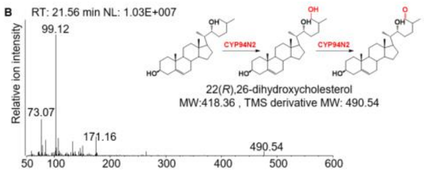	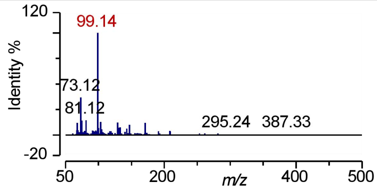
22-hydroxy-26-aminocholesterol[1, 3]	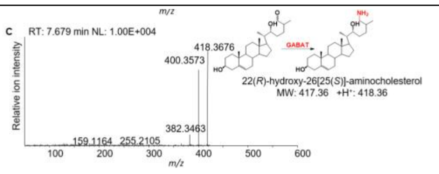	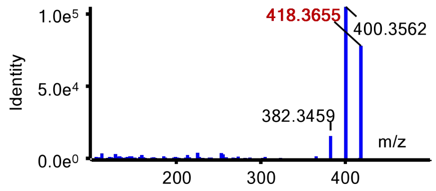
Verazine[1,3]	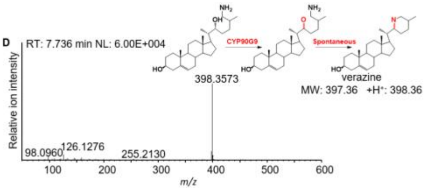	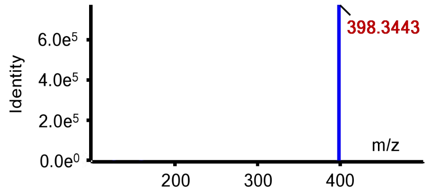

[1] Zhou, C., Yang, Y., Tian, J., Wu, Y., An, F., Li, C., Zhang, Y. (2022), 22R- but not 22S-hydroxycholesterol is recruited for diosgenin biosynthesis. *Plant J.* 109: 940–951.

[2]Kou, C., Liu, J., Yin, X., He, D., Liu, J., Hua, X., Ma, R., Sun, W., Xue, Z., Ma, P. (2024) Efficient heterologous biosynthesis of verazine, a metabolic precursor of the anti-cancer drug cyclopamine. *Plant Commun*. *5*: 100831.

[3] Augustin, M.M., Shukla, A.K., Starks, C.M. Linna Han, M., Holland, C.K., Kutchan, T.M. (2017) Biosynthesis of Veratrum californicum specialty chemicals in Camelina sativa seed. *Plant Biotechnol Rep*. 11: 29–41.
