# Supplementary material for: Reconstruction of de novo verazine biosynthesis in Saccharomyces cerevisiae and computational analysis of cholesterol-binding geometry for 22R hydroxylation by VcCYP90B27 to dissect the cyclopamine biosynthetic pathway
Source: Eng Life Sci. 2026 Aug 12;26(8):e008. doi: 10.48130/els-0026-0008 (PMC13599646; doi:10.48130/els-0026-0008)
Supplement: Supplementary file 1 [file els-2026-26-8-0008-S1.zip › 10.48130_els-0026-0008-Suppl-TableS5.pdf]

Table S5 Sequence of each gene used in the study

ERG1
ATGTCTGCTGTTAACGTTGCACCTGAATTGATTAATGCCGACAACACAATTACCTACGA<br>TGCGATTGTCATCGGTGCTGGTGTTATCGGTCCATGTGTTGCTACTGGTCTAGCAAGAA<br>AGGGTAAGAAAGTTCTTATCGTAGAACGTGACTGGGCTATGCCTGATAGAATTGTTGGT<br>GAATTGATGCAACCAGGTGGTGTTAGAGCATTGAGAAGTCTGGGTATGATTCAATCTAT<br>CAACAACATCGAAGCATATCCTGTTACCGGTTATACCGTCTTTTTCAACGGCGAACAAG<br>TTGATATTCCATACCCTTACAAGGCCGATATCCCTAAAGTTGAAAAATTGAAGGACTTG<br>GTCAAAGATGGTAATGACAAGGTCTTGGAAGACAGCACTATTCACATCAAGGATTACG<br>AAGATGATGAAAGAGAAAGGGGTGTTGCTTTTGTTCATGGTAGATTCTTGAACAACTT<br>GAGAAACATTACTGCTCAAGAGCCAAATGTTACTAGAGTGCAAGGTAACTGTATTGAG<br>ATATTGAAGGATGAAAAGAATGAGGTTGTTGGTGCCAAGGTTGACATTGATGGCCGTG<br>GCAAGGTGGAATTCAAAGCCCACTTGACATTTATCTGTGACGGTATCTTTTCACGTTTC<br>AGAAAGGAATTGCACCCAGACCATGTTCCAACTGTCGGTTCTTCGTTTGTCGGTATGTC<br>TTTGTTCAATGCTAAGAATCCTGCTCCTATGCACGGTCACGTTATTCTTGGTAGTGATCA<br>TATGCCAATCTTGGTTTACCAAATCAGTCCAGAAGAAACAAGAATCCTTTGTGCTTACA<br>ACTCTCCAAAGGTCCCAGCTGATATCAAGAGTTGGATGATTAAGGATGTCCAACCTTTC<br>ATTCCAAAGAGTCTACGTCCTTCATTTGATGAAGCCGTCAGCCAAGGTAAATTTAGAGC<br>TATGCCAAACTCCTACTTGCCAGCTAGACAAAACGACGTCACTGGTATGTGTGTTATCG<br>GTGACGCTCTAAATATGAGACATCCATTGACTGGTGGTGGTATGACTGTCGGTTTGCAT<br>GATGTTGTCTTGTTGATTAAGAAAATAGGTGACCTAGACTTCAGCGACCGTGAAAAGG<br>TTTTGGATGAATTACTAGACTACCATTTCGAAAGAAAGAGTTACGATTCCGTTATTAAC<br>GTTTTGTCAGTGGCTTTGTATTCTTTGTTCGCTGCTGACAGCGATAACTTGAAGGCATTA<br>CAAAAAGGTTGTTTCAAATATTTCCAAAGAGGTGGCGATTGTGTCAACAAACCCGTTG<br>AATTTCTGTCTGGTGTCTTGCCAAAGCCTTTGCAATTGACCAGGGTTTTCTTCGCTGTC<br>GCTTTTTACACCATTTACTTGAACATGGAAGAACGTGGTTTCTTGGGATTACCAATGGC<br>TTTATTGGAAGGTATTATGATTTTGATCACAGCTATTAGAGTATTCACCCCATTTTTGTTT<br>GGTGAGTTGATTGGTTAA
*Dr*DHCR7
ATGATGGCTTCTGATAGAGTTAGAAAAAGACATAAAGGTTCTGCTAATGGTGCTCAAAC<br>TGTTGAAAAAGAACCATCTAAAGAACCTGCTCAATGGGGTAGAGCTTGGGAAGTTGAT<br>TGGTTTTCTTTGTCTGGTGTGATTTTGTTGTTGTGTTTTGCTCCATTTTTGGTATTTTTCT<br>TTATAATGGCTTGTGATCAATATCAATGTTCTATTTCTCATCCATTGTTGGATTTATATAAT<br>GGTGATGCTACTTTGTTTACTATTTGGAATAGAGCTCCATCTTTTACTTGGGCTGCTGCT<br>AAAATTTATGCTATTTGGGTTACTTTTCAAGTTGTTTTGTATATGTGTGTTCCTGATTTTT<br>TGCATAAAATTTTGCCTGGTTATGTTGGTGGTGTTCAAGATGGTGCTAGAACTCCTGCT<br>GGTTTGATTAATAAATATGAAGTTAATGGTTTGCAATGTTGGTTGATTACTCATGTTTTGT<br>GGGTTTTGAATGCTCAACACTTCCATTGGTTTAGTCCAACGATAATTATTGATAATTGGA<br>TTCCATTGTTGTGGTGTACTAATATCTTGGGTTACGCGGTTTCAACTTTCGCATTTATCA<br>AGGCTTATTTGTTCCCTACTAATCCTGAAGATTGCAAATTTACTGGCAATATGTTTTATAA<br>TTATATGATGGGAATCGAATTTAACCCGCGCATCGGTAAATGGTTTGATTTCAAATTGTT<br>TTTTAATGGTAGACCTGGTATTGTTGCTTGGACTTTGATTAATTTGTCTTACGCTGCCAA<br>ACAACAAGAACTCTATGGGTATGTTACGAATTCTATGATTTTGGTTAATGTTTTGCAAGC<br>TGTTTACGTTGTTGATTTCTTTTGGAACGAAGCTTGGTATTTGAAAACTATTGATATTTG

TCATGATCATTTTGGTTGGTATTTGGGTTGGGGTGATTGTGTTTGGTTGCCATTTTTGTAT<br>ACTTTGCAAGGTTTGTATTTGGTTTATAATCCAATTCAATTGTCTACTCCACATGCTGCT<br>GGCGTATTGATTTTGGGTCTAGTTGGTTACTATATTTTCAGAGTTACTAATCATCAAAAG<br>GACTTGTTTAGAAGAACTGAAGGTAATTGTTCTATTTGGGGTAAAAAACCAACTTTTAT<br>TGAATGTTCTTATAGATCTGCTGATGGTGCTATTCATAAATCTAAATTGATGACTTCTGGT<br>TTTTGGGGTGTTGCTAGACATATGAATTATACTGGTGATTTGATGGGTTCTTTGGCTTATT<br>GTTTGGCTTGTGGAGGTAACCATCTATTGCCGTACTTCTATATTGTTTACATGACTATTTT<br>GTTGGTTCATAGATGTATTAGAGATGAACATAGATGTTCTAATAAATATGGTAAAGATTG<br>GGAAAGATATACTGCTGCTGTTTCTTATAGATTGTTGCCAAATATTTTTTAA
*Gg*DHCR24
ATGTCTGCTGTTTGGTCTTTGGGTGCTGGTTTGTTATTGTTATTGTTGTGGGTTAGACAT<br>AGAGGTTTGGAAGCTGTTTTGGTTCATCACAGATGGATTTTTGTCTGTTTTTTTTTGATG<br>CCATTGTCTATTTTGTTTGATGTTTATTATCAATTGAGAGCTTGGGCTGTTAGAAGAATG<br>CATTCTGCTCCAAGATTGCATGGTCAAAGAGTTAGACATATTCAAGAACAAGTTAGAG<br>AATGGAAAGAAGAAGGTGGTAGAAGATATATGTGTACTGGTAGACCTGGTTGGTTGAC<br>TGTTTCTTTAAGGGTTGGTAAGTACAAGAAGACTCATAAAAATATTATGATTAATTTGAT<br>GGATGTTTTGGAAGTTGATTCTGAAAGACAAGTTGTTAGAGTTGAACCATTGGTTACTA<br>TGGGTCAATTGACTGCTTATTTGAATCCAATGGGTTGGACTATTCCTGTTGTTCCTGAAT<br>TGGATGATTTGACTGTTGGTGGTTTGATTATGGGTACTGGTATTGAATCTTCATCTCATAT<br>TTATGGTTTGTTTCAACATACTTGTATGGCTTATGAATTGGTTTTGGCTGACGGTTCTTT<br>GGTCAGATGTTCTCCAACTGAAAATTCTGATTTGTTTTATGCTGTTCCATGGTCTTGTGG<br>TACTTTGGGTTTCTTAGTTGCAGCAGAGATTAAAATGATTCCTGCTAAAAAATATATCAG<br>ATTGCATTATGAACCTGTTAGAGGTTTGAGGTCTATTTGCGAGAAGTTTACTGAAGAAT<br>CTAAAAATAAAGAAAATTCATTTGTTGAAGGTTTGGTTTATTCTTTGGAAGAAGCTGTT<br>ATTATGACTGGTGTTTTGACTGACGAAGCAGAACCTTCAAAGATCAACAGAATTGGTA<br>ATTACTACAAGCCTTGGTTTTTTAAACATGTCGAAAAATACTTAAAAGCAAACAAAACT<br>GGTATTGAATATATTCCTTCTAGACACTATTATCATAGACATACTAGATCTATCTTTTGGG<br>AATTGCAAGATATTATTCCATTTGGTAATAATCCTGTTTTTAGATATTTGTTTGGTTGGAT<br>GGTTCCACCAAAAATTTCTTTGTTGAAATTGACTCAAGGTGAAGCTATTAGAAAATTGT<br>ATGAACAACATCATGTCGTTCAAGATATGTTAGTCCCAATGAAATCTTTGGAGAAGTCT<br>ATCCAAACTTTTCATGTTGATTTGAATGTTTATCCATTGTGGTTGTGTCCATTTTTGTTGC<br>CAAATAATCCTGGTATGGTTCATCCAAAAGGTGATGAAACTGAATTGTATGTTGATATTG<br>GTGCTTATGGTGAACCAAAAACTAAACAATTTGAAGCTAGAGCTTCTATGAGACAAAT<br>GGAAAAATTTGTTAGATCTGTTCATGGTTTTCAAATGTTGTATGCTGATTGTTATATGACT<br>AGAGAAGAATTTTGGGATATGTTTGATGGTTCTTTGTATCATTCTTTGAGAGAACAAAT<br>GAATTGTAAAGATGCTTTTCCTGAAGTTTATGATAAAATTTGTAAAGCTGCTAGACATTA<br>A
*Vv*CPR
ATGCAATCATCCTCCGTAAAGGTATCCCCATTCGACTTAATGTCAGCAATCATCAAGGGT<br>TCTATGGACCAATCAAACGTATCATCAGAATCAGGTGGTGCTGCAGCCATGGTTTTGGA<br>AAACAGAGAATTCATTATGATCTTGACTACATCCATTGCTGTTTTGATCGGTTGTGTTGT<br>CGTATTGATATGGAGAAGATCAGGTCAAAAACAATCCAAGACTCCAGAACCACCTAAA<br>CCTTTGATTGTTAAGGATTTGGAAGTAGAAGTTGATGACGGTAAACAAAAGGTTACAAT<br>ATTTTTCGGTACACAAACCGGTACTGCTGAAGGTTTCGCAAAAGCCTTGGCTGAAGAA<br>GCAAAGGCCAGATACGAAAAGGCAATTTTTAAGGTTGTCGATTTGGATGACTATGCCG<br>GTGACGACGATGAATACGAAGAAAAATTGAAAAAGGAAACTTTGGCCTTTTTCTTTTT

GGCTACATATGGTGACGGTGAACCAACCGACAATGCTGCAAGATTCTACAAATGGTTT<br>GCTGAGGGTAAAGAACGTGGTGAATGGTTGCAAAACTTAAAGTATGGTGTTTTCGGTT<br>TGGGTAACAGACAATACGAACATTTCAACAAAGTTGCAAAGGTAGTTGACGATATAAT<br>CACAGAACAAGGTGGTAAAAGAATCGTCCCAGTAGGTTTGGGTGACGATGACCAATGT<br>ATTGAAGATGACTTCGCCGCTTGGAGAGAATTATTATGGCCTGAATTAGATCAATTGTTA<br>AGAGACGAAGATGACGCTACCACTGTATCTACACCATATACCGCAGCCGTTTTGGAATA<br>CAGAGTCGTATTTCATGATCCTGAAGGTGCATCATTACAAGACAAGTCATGGGGTTCCG<br>CCAATGGTCATACTGTTCACGATGCTCAACACCCATGTAGAGCCAACGTTGCTGTCAGA<br>AAAGAATTGCATACTCCTGCTAGTGATAGATCTTGCACACACTTGGAATTCGACATTTC<br>TGGTACTGGTTTAACATATGAAACCGGTGACCATGTAGGTGTTTACTGTGAAAATTTGC<br>CAGAAACAGTCGAAGAAGCAGAAAGATTGTTAGGTTTCTCACCTGATGTATATTTTTCC<br>ATACACACCGAAAGAGAAGACGGTACTCCATTAAGTGGTTCTTCATTGTCTCCACCTTT<br>TCCACCTTGCACTTTGAGAACAGCATTAACCAGATACGCCGATGTTTTGTCCAGTCCTA<br>AAAAGTCTGCATTGGTCGCCTTAGCTGCACATGCATCAGATCCATCCGAAGCCGACAG<br>ATTGAAATATTTGGCTAGTCCTTCTGGTAAAGATGAATACGCTCAATGGGTTGTCGCAA<br>GTCAAAGATCTTTGTTAGAAATTATGGCCGAATTTCCATCTGCTAAGCCACCTTTGGGT<br>GTCTTCTTTGCCGCTGTAGCTCCAAGATTGCAACCTAGATACTACAGTATCTCTTCATCC<br>CCAAAGATGGTTCCTTCTAGAATACATGTTACCTGTGCATTGGTCTGCGATAAAATGCC<br>AACTGGTAGAATCCACAAGGGTATTTGTTCAACATGGATGAAATATGCCGTTCCATTAG<br>AAGAATCACAAGATTGCTCCTGGGCACCTATCTTCGTTAGACAATCAAACTTCAAATTG<br>CCAGCTGATACCTCCGTCCCTATCATTATGATTGGTCCAGGTACAGGTTTAGCTCCTTTC<br>AGAGGTTTCTTGCAAGAAAGATTTGCATTGAAGGAAGCTGGTGCAGAATTGGGTAGTT<br>CTATCTTGTTCTTTGGTTGTAGAAACAGAAAGATGGATTACATCTACGAAGACGAATTG<br>AACGGTTTCGTAGAAAGTGGTGCTTTGTCTGAATTGATCGTTGCATTTTCAAGAGAAG<br>GTCCAACTAAGGAATACGTTCAACATAAGATGATGGAAAAGGCTAGTGATATCTGGAA<br>CGTCATCTCTCAAGGTGGTTATATATACGTATGCGGTGACGCTAAGGGTATGGCAAGAG<br>ACGTTCATAGAACTTTGCACACAATCTTACAAGAACAAGGTTCTTTAGATTCATCCAAG<br>GCTGAATCAATGGTAAAGAACTTACAAATGACTGGTAGATACTTGAGAGATGTCTGGTA<br>A
*Vc*CYP90B27
ATGGCTATGGAATTGTTGTTGTTGATCCCAGCTTTTATTGTTGCAATCATCATTTTCTTTT<br>CTTTTAAATCAACTAACGGTACTTCTACAAAGCCATTGAAGTTGCCTCCAGGTCAAATG<br>GGTTGGCCTTTTATTGGTCATACAATCCCTTTTATGCAACCACATTCTTCAGCTTCTTTG<br>GGTCCATACATCGATTTGAACACTGCAAGATACGGTACAATTTTTAGAATGAATTTGTTG<br>GCTAAGCCAACTATCGTTTCAGCAGATCCAGAATTCAATAGATACATCTTGCAAAACGA<br>AGGTAGATTGTTCGAAAACTCTTGTCCAACTTCAATTGCTGAAATTATGGGTAGATGGT<br>CTATGTTGGCATTAACAGGTGACGTTCATAGAGAAATGAGATCAATCGCTGTTTCTTTTA<br>TGTCAAACGTTAAGTTGAGAACATACTTCATCGGTGACATCGAACAACAAGCAATTAA<br>AGTTTTGGCTTCTTGGGCAGGTAGAGATGCTCCATTTTCAGCACAAGATGAAGGTAAA<br>AAGTTCGCTTTTAATTTGATGGTTAAACATTTGATGTCTATGGAACCAGGAATGAAGGA<br>AACTGAACAATTGAGATCTGAATACCATGCTTTTATGAAGGGTATGGCATCAATTCCAAT<br>TAATTTGCCAGGTACAGCTTACAGAAAAGCATTGCAATCTAGATCAATCATCTTGAAGA<br>TTATGGGTGAAAAATTAGATGAAAGAATTAAACAAGTTAAAGAAGGTTGTGAAGGTTT<br>GGAACAAGATGATTTGTTAGCTTCTGTTTCAAAGCATCCAAATTTGGCAAAGGAACAA<br>ATCTTGGATTTGATCTTGTCTATGTTGTTTGCTGGTCATGAAACTTCTTCAGCTGCAATC<br>GCTTTGGCAATATACTTTTTGGAATCATGTCCAAAAGCTGTTGAACAATTGAGAGAAGA<br>ACATAAGGAAATCGCAAGACAAAAGAAAGAAAGAGGTGAAACAGGTTTGAACTGGG<br>ATGATTACAAGAAAATGGAATTCACTCATTGTGTTATTAATGAAACATTGAGAATGGGT

AACATCGTTAAGTTCTTACATAGAAGAGCTATTAAAGATGTTCAATTCAAAGGTTACGAT<br>ATCCCATGTGGTTGGGAAGTTGTTCCAATTATTTCTGCTGCACATTTGGATTCTTCAATC<br>TATGATGATCCACAAAGATACGATCCATGGAGATGGCAAGCTATTTTAGCTGGTAACAC<br>TAAAAATAACAACGTTACATCAATTATGTCTTTTTCAGGTGGTCCAAGATTGTGTCCAG<br>GTGCTGAATTGGCAAAGTTGGAAATCGCTGTTTTCTTGCATCATTTGGTTCAAAAGTAC<br>CAATGGGAAATGGCAGAACATGATTACCCAGTTTCTTTCCCATTTTTAGGTTTCCCAAA<br>GAGATTGCCAATTAAAGTTAGACCATTGGGTGACTAA
*Vc*CYP94N1
ATGGATTTGCCATCTGCTTCAGCTGCTGTTGCTGCTGCTACTGCTGCAGTTATTTTCTTG<br>TTGACTATCTACCTGCTGCCAAAGAAAAAATCTCCAGCTTCTACTGGTAAGAACGGTTC<br>TACTTCTTTGGAATCTTACCCAGTTATTGGTAACTTGCCACACTTCGTTAAGAACAGAA<br>ACAGATTCTTGGATTGGGTTGCCGAAATCATTTCTCAATCTCCAACTGGTACTGTTATTG<br>CTGCTCCATTGGTTTTTACTTCTAACCCAGAAAACGTTGAACATACCGCTAAGTCTAGA<br>TTTGATGCTTATGCTAGAGGTCCAGCTGCTACAGCTGTTTTACATGATTTTTTAGGTTCC<br>GGCATCTTGAACGTTGATGGTGATAGTTGGAGGGCTCAAAGAAAGACTGCTTCTTCTG<br>AATTCACTACCAGATCTTTGAGAGCCTTCATTTTGGATGCTGTTGACGGTGAAGCTGCT<br>GGTAGATTATTGCCATTATTGTCTAGAGCTGCTGCTTCTGGTGAAGTTTTTGACTTGCAA<br>GATGTCTTGGAAAGATTCGCCTTCGATAACATTTGCTCCATTATTTCGATGCCGATCCA<br>AACTGTTTGAACGATACTCATGATGGTGTTGGTGAAAGATTCTACCATGCTTTTCATGAT<br>GCTACCTTGTTGTCTACTGGCAGATATTACTATCCATTCCATTGGGTTTGGAGGTTGTTG<br>AGATGGTTGAATTTGGGTACTGAAAAGCGTTTGAGAGATGCCGTTTCTGATGTTCATAA<br>GGCCATTGATGAATTGGTCGGTTCTAGAAAAACTGAAGTTGGTACTACTGTTAGAAGG<br>CAAGGTGGTGGTTCTGATTTGTTGTCAAGATTTGCTGAAGGTGGTGATTACTCCGATGA<br>TGTTTTAAGGGATGTCTTGATCAACTTCGTTTTGGCTGGTAGAGATACAACTCCATCAG<br>CTTTGACTTGGTTTTTCTTTATGATCTCCTCCAGACCAGATGTTGTTGATCAAATTTTGG<br>ACGAGATCAGGTCCATCAGAGATCATCAAGATAGATCTAATCCAAACGGTGGTGGTGG<br>CGGTTTTACTTTGGAAGAATTGAGAGAAATGAACTACTTGCATGCTGCCATTACCGAAT<br>CCTTGAGATTGAATCCACCAGTTCCATTGATGCCAAAGATGTGTATGGAAGATGATGTA<br>TTGCCAGATGGTACTGTAGTTAGAAGAGGTTGGACTGTTATGTACTCTGCTTTTGCTATG<br>GGTAGAAAGGCTGAAATTTGGGGTGAAGATTGCATGGAATTCAAACCAGAAAGATGGT<br>TGGATGATGGTGGTTGTTTTAAATCTGCTTCCGCTTATAGATTGCCAGCATTTCATGCTG<br>GTCCTAGAATTTGTTTGGGTAAAGATATGGCCTACATTCAGATGAAGGCTGTTGCTTCAT<br>CTTTGTTGGAAAGGTTCGAAGTTGAAGTCGTTGAAAAAAGAGGTAAGCCAGAATTGT<br>CCATCACCATGAGAATGGACAGAGGTTTGCCAGTTAGAGTGAAAGAAAGAAAGCGTG<br>GTTGTTAA
*Vc*GABAT
ATGGGATCCACTGAGGCGCCTGTATCAACTCCCAACCCCGGCGTACAGACAGTTGCGG<br>CGAACGGGTCATCATGCTTGGCAGGGACCAAGTCCGTTGAAGCGAAAGGGTTTAAGG<br>GGCATGACATGCTGGCACCTTTCACCCCTGGATGGAAGAGCACCTCCTTGGAGCCTGT<br>TGTCATTGAGAAGTCGGAGGGTTGTTATGTTTACGACAATCATGGAACGAAATATCTTG<br>ATACTATTGCTGGGTTGTGGTGCACGTCTTTAGGAGGAAATGAGCCTCGACTTATAGAA<br>GCTGCAACTGCACAGTTAAATAAATTGCCATTTTACCACAACTTCTGCAATCGTGTCAC<br>CATACCCTCTTTGGATCTAGCCAAAGAACTTCTTGATATGTTCACTGCCAAGAAAATGG<br>GAAAGGTGTTCTTCACTAATAGTGGTTCTGAGGCCAATGACACTCAGGTGAAGCTGGT<br>TCGATATTATTTCAATGCACTGGGAAAGCCAAATAAGAAGAAATTTATAGCTCAAACGA<br>AATCGTTCCATGGAACTACAGTAGCATCTGCTAGTCTCTCGGGCCTTATGCATCTACATT<br>ATAAGTTCGATCTGCCAATTCCAAATGTATTGCACTCAGATTGTCCACACTATTGGCGTT

ACCATCTACCAGGTGAGTCGGAAGAGGAGTTCTCGTCAAGGTTAGCTGATAATTTGGA<br>GAAACTTATTCTAAAAGAAGAACCAGATACGGTTGCTGCTTTCATTGCTGAACCTGTCA<br>TGGGCGCATCGGGTGTTTTCCTTCCCCCCGAGACCTACTTTGAGAAGATTCAAGCTGTC<br>CTAAAGAAATACGATATTCTCTTCATTGCGGATGAGGTCGTCACTGCATTCGCAAGGTT<br>AGGGACAATGTTTGGATGTGACAAATACAACATTCAGCCGGACCTCGTCTCCTTAGCA<br>AAAGCTCTTTCATCTGCCTACATTCCCATCGGCGCAGTTATCGTTAGCCAAGAAATTTCT<br>GAAGTTATAAATCGTCAAAGCACCCAAATTGGTACATTTGCTCACGGGTGTACGTTTTC<br>GGGGCATCCAGTGGCCTGCGCTGTTGCTTTGGAGACACTTAAGATTTATAAGGAGAGG<br>AATATTGTTGAGCGCGTCCAGGCTATATCAAAAAGGTTTCAGGATGGCATCAAAGCCTT<br>CTCTGATAGCCCCATTATTGGGGAGATTCGTGGAACCGGGTTGATCATCGGGGTGGACT<br>TTACTAAGAACAAGTCTCCGAATGATGTCTTCCCTTACAAGTGGGGGGTCGGAGGAAT<br>ATTTGTATCAGAATGCGCAAAACGCGGGATGATAGTTCATGAGCCTATCGGAGATTGCA<br>CGACGTTGTCACCTGCACTGATAATATCTGAAGAAGAGATTGAACAAATAATACAAATC<br>TTTGGAGAAGCCCTGAAGAGTACAGAGAAGCAAGTGGAGCAGCTCCAGTCTCAGAAT<br>ACCGCAACTTAA
*Vc*CYP94G1
ATGACTCCATTAGTTGTTCTCTTCTTTCTGTTCCCTACACTACTAGTGTTGGTGGTTGCA<br>GCATTTGGGCAGCTAGCAGGCAAGGATGATGGGTGGAGGAAGAGGGGGCTGAGGCTC<br>CCACCGGGCACCATGGGTTGGCCGCTCGTCGGAGAAACCCTGTCCTTCGGAAAGATCC<br>ACCCCAGCACTTCCATCGGAGACTACCTCGAGGAGCACATCCACAAGTACGGAAAGAT<br>TTTCAAGGCGAACTTGTTCGCATCTCAGGCGGTGGTTTCGGTGGACGCGGAGCTGAAC<br>CGGTTCGTGATGCTGAATGACGGGCGGCTGTTCGAGCCGTGCACCCCGAAGGGGGTG<br>CTGGACATCCTGGGGCACGCGACGCCGATGGCGTTGTCGGGTGATCTGCACCGCTACA<br>TCAAGTCTCTGTCCGTTGATTTCATGGGGATCGGAAGGATGAAGAGCTACTTCCTCCCC<br>GACGCCGAGCGGTACATCACGGAGACGCTCGCCTCGTGGGAGGAGGGCACGCCATTC<br>CAAGCCAAGGAGGAGGCATCCAAGATGATGTTCAATTTGATGGTGAAGAACGTTCTCA<br>GCATGAAAGCTGGTGTCCCCGAGACTGAGCGGCTCCGCAAGCTTTACATGTCTTTCAT<br>GAAGGGGGTCATTGCATTACCTCTCAATTTCCCTGGATCTGCCTACAAAAAAGCCGTAG<br>AGGCAAGAAAAGTGATTCTGGGAGTGATAAACGAGTTGATGAAGGAAAGGATCCAAA<br>AGAGAAGAGACGGAACGGACGATATCGGCGAAGCTGACCTATTAGGGTTCGTACTCGA<br>GCAGTCCAACCTCGACGCCGAGCAGTTTGGCGATCTCTTGTTGGGTTTGTTGTTCGGC<br>GGCCACGAGACTTCAGCTACGGCCATCACCCTGCTCATCTACTTCCTTCACGACTGCCC<br>TTTAGCCGTTAAACAACTCCGGGAAGAGCACATGGAGATCGTGAGGATGAAAAGGCA<br>AAGAGGAGAGCCTGCTGCACTAACATGGGAGGACTACAAACTGATGGAGTTTAGCCA<br>ATGTGTGGTGCGAGAGACTCTTCGATTGGGTAACGTGGTCAAGTTTATTGTGCGCAAG<br>GCGAGCACTGATATTAAATTCAAAGGGTATGATATTCCCAAAGGGTGGACCGTGTTGCC<br>GATCTTAACAGCCGCCCATGTTGATCCCTCTGTTTATGAGAACGTTCACAAATTCGATC<br>CATGGAGATGGCAGACTGGTTCTACAAGCAAAGCCTTGAACGACAACTACATGCCTTT<br>CGGTTTGGGATTGCGCAACTGCGCGGGCCTGCAACTCGCCAAGTTGGAGATTGTTGTG<br>TTTCTTCACCATCTCGTACTCAACTTCGACTGGGAGCTGGCAGAGCCCGACAATCCCAT<br>AGCGTCCCCTTTCCCCGAGTTCCCCAGGGGCCTACCCATCAAGGTTCGCCGGCTTTCG<br>CTCCTCCAATAA
TEF1 promoter
AAGTGATCCCCCACACACCATAGCTTCAAAATGTTTCTACTCCTTTTTTACTCTTCCAGA<br>TTTTCTCGGACTCCGCGCATCGCCGTACCACTTCAAAACACCCAAGCACAGCATACTA<br>AATTTCCCCTCTTTCTTCCTCTAGGGTGTCGTTAATTACCCGTACTAAAGGTTTGGAAAA<br>GAAAAAAGAGACCGCCTCGTTTCTTTTTCTTCGTCGAAAAAGGCAATAAAAATTTTTAT

CACGTTTCTTTTTCTTGAAAATTTTTTTTTTTGATTTTTTTCTCTTTCGATGACCTCCCAT
TGATATTTAAGTTAATAAACGGTCTTCAATTTCTCAAGTTTCAGTTTCATTTTTCTTGTTC
TATTACAACTTTTTTTACTTCTTGCTCATTAGAAAGAAAGCATAGCAATCTAATCTAAGT
TTTAATTACAAA

TDH3 promoter

ATACTAGCGTTGAATGTTAGCGTCAACAACAAGAAGTTTAATGACGCGGAGGCCAAGG
CAAAAAGATTCCTTGATTACGTAAGGGAGTTAGAATCATTTTGAATAAAAAACACGCTT
TTTCAGTTCGAGTTTATCATTATCAATACTGCCATTTCAAAGAATACGTAAATAATTAATA
GTAGTGATTTTCCTAACTTTATTTAGTCAAAAAATTAGCCTTTTAATTCTGCTGTAACCC
GTACATGCCCAAAATAGGGGGCGGGTTACACAGAATATATAACATCGTAGGTGTCTGGG
TGAACAGTTTATTCCTGGCATCCACTAAATATAATGGAGCCCGCTTTTTAAGCTGGCATC
CAGAAAAAAAAAGAATCCCAGCACCAAAATATTGTTTTCTTCACCAACCATCAGTTCAT
AGGTCCATTCTCTTAGCGCAACTACAGAGAACAGGGGCACAAACAGGCAAAAAACGG
GCACAACCTCAATGGAGTGATGCAACCTGCCTGGAGTAAATGATGACACAAGGCAATT
GACCCACGCATGTATCTATCTCATTTTCTTACACCTTCTATTACCTTCTGCTCTCTCTGAT
TTGGAAAAAGCTGAAAAAAAAGGTTGAAACCAGTTCCCTGAAATTATTCCCCTACTTG
ACTAATAAGTATATAAAGACGGTAGGTATTGATTGTAATTCTGTAAATCTATTTCTTAAAC
TTCTTAAATTCTACTTTTATAGTTAGTCTTTTTTTTAGTTTTAAAACACCAAGAACTTAGT
TTCGAATAAACACACATAAACAAACAAA

FBA1 promoter

GCACCGCTGGCTTGAACAACAATACCAGCCTTCCAACTTCTGTAAATAACGGCGGTAC
GCCAGTGCCACCAGTACCGTTACCTTTCGGTATACCTCCTTTCCCCATGTTTCCAATGCC
CTTCATGCCTCCAACGGCTACTATCACAAATCCTCATCAAGCTGACGCAAGCCCTAAGA
AATGAATAACAATACTGACAGTACTAAATAATTGCCTACTTGGCTTCACATACGTTGCAT
ACGTCGATATAGATAATAATGATAATGACAGCAGGATTATCGTAATACGTAATAGTTGAA
AATCTCAAAAATGTGTGGGTCATTACGTAAATAATGATAGGAATGGGATTCTTCTATTTT
TCCTTTTTCCATTCTAGCAGCCGTCGGGAAAACGTGGCATCCTCTCTTTCGGGCTCAAT
TGGAGTCACGCTGCCGTGAGCATCCTCTCTTTCCATATCTAACAACTGAGCACGTAACC
AATGGAAAAGCATGAGCTTAGCGTTGCTCCAAAAAAGTATTGGATGGTTAATACCATTT
GTCTGTTCTCTTCTGACTTTGACTCCTCAAAAAAAAAAAAATCTACAATCAACAGATCGC
TTCAATTACGCCCTCACAAAAACTTTTTTCCTTCTTCTTCGCCCACGTTAAATTTTATCC
CTCATGTTGTCTAACGGATTTCTGCACTTGATTTATTATAAAAAGACAAAGACATAATAC
TTCTCTATCAATTTCAGTTATTGTTCTTCCTTGCGTTATTCTTCTGTTCTTCTTTTTCTTTT
GTCATATATAACCATAACCAAGTAATACATATTCAAA

PGK1 promoter

TGACTTCCCATACTGTAATTGCTTTTAGTTGTGTATTTTTAGTGTGCAAGTTTCTGTAAAT
CGATTAATTTTTTTTTCTTTCCTCTTTTTATTAACCTTAATTTTTATTTTAGATTCCTGACT
TCAACTCAAGACGCACAGATATTATAACATCTGCATAATAGGCATTTGCAAGAATTACTC
GTGAGTAAGGAAAGAGTGAGGAACTATCGCATACCTGCATTTAAAGATGCCGATTTGG
GCGCGAATCCTTTATTTTGGCTTCACCCTCATACTATTATCAGGGCCAGAAAAAGGAAG
TGTTTCCCTCCTTCTTGAATTGATGTTACCCTCATAAAGCACGTGGCCTCTTATCGAGAA
AGAAATTACCGTCGCTCGTGATTTGTTTGCAAAAAGAACAAAACTGAAAAAACCCAG
ACACGCTCGACTTCCTGTCTTCCTATTGATTGCAGCTTCCAATTTCGTCACACAACAAG
GTCCTAGCGACGGCTCACAGGTTTTGTAACAAGCAATCGAAGGTTCTGGAATGGCGGG
AAAGGGTTTAGTACCACATGCTATGATGCCCACTGTGATCTCCAGAGCAAAGTTCGTTC
GATCGTACTGTTACTCTCTCTCTTTCAAACAGAATTGTCCGAATCGTGTGACAACAACA

GCCTGTTCTCACACACTCTTTTCTTCTAACCAAGGGGGTGGTTTAGTTTAGTAGAACCT
CGTGAAACTTACATTTACATATATATAAACTTGCATAAATTGGTCAATGCAAGAAATACA
TATTTGGTCTTTTCTAATTCGTAGTTTTTCAAGTTCTTAGATGCTTTCTTTTTCTCTTTTTT
ACAGATCATCAAGGAAGTAATTATCTACTTTTTACAACAAATATAAAACA

ADH1 terminator

GCGAATTTCTTATGATTTATGATTTTTATTATTAAATAAGTTATAAAAAAAATAAGTGTAT
ACAAATTTTAAAGTGACTCTTAGGTTTTAAAACGAAAATTCTTATTCTTGAGTAACTCTT
TCCTGTAGGTCAGGTTGCTTTCTCAGGTATAGCATGAGGTCGCTC

CYC1 terminator

ATCCGCTCTAACCGAAAAGGAAGGAGTTAGACAACCTGAAGTCTAGGTCCCTATTTATT
TTTTTATAGTTATGTTAGTATTAAGAACGTTATTTATATTTCAAATTTTTCTTTTTTTTCTGT
ACAGACGCGTGTACGCATGTAACATTATACTGAAAACCTTGCTTGAGAAGGTTTTGGG
ACGCTCGAAG

ENO2 terminator

AGTGCTTTTAACTAAGAATTATTAGTCTTTTCTGCTTATTTTTTCATCATAGTTTAGAACA
CTTTATATTAACGAATAGTTTATGAATCTATTTAGGTTTAAAAATTGATACAGTTTTATAA
GTTACTTTTTCAAAGACTCGTGCTGTCTATTGCATAATGCACTGGAAGGGGAAAAAAA
AGGTGCACACGCGTGGCTTTTTCTTGAATTTGCAGTTTGAAAAAT

FBA1 terminator

GTTAATTCAAATTAATTGATATAGTTTTTTAATGAGTATTGAATCTGTTTAGAAATAATGG
AATATTATTTTTATTTATTTATTTATATTATTGGTCGGCTCTTTTCTTCTGAAGGTCAATGA
CAAAATGATATGAAGGAAATAATGATTTCTAAAATTTTACAACGTAAGATATTTTTACAA
AAGCCTAGCTCATCTTTTGTCATGCACTATTTTACTCACGCTTGAAATTAACGGCCAGTC
CACTGCGGAGTCATTTCAAAGTCATCCTAATCGATCTATCGTTTTTGATAGCTCATTTTG
GAGTTCGCGATTGTCTTCTGTTATTCACAACTGTTTTAATTTTTATTTCATTCTGGAACTC
TTCGAGTTCTTTGTAAAGTCTTTCATAGTAGCTTACTTTATCCTCCAACAT

PDC1 terminator

GCGATTTAATCTCTAATTATTAGTTAAAGTTTTATAAGCATTTTTATGTAACGAAAAATAA
ATTGGTTCATATTATTACTGCACTGTCACTTACCATGGAAAGACCAGACAAGAAGTTGC
CGACAGTCTGTTGAATTGGCCTGGTTAGGCTTAAGTCTGGGTCCGCTTCTTTACAAATT
TGGAGAATTTCTCTTAAACGATATGTATATTCTTTTCGTTGGAAAAGATGTCTTCCAAAA
AAAAAACCGATGAATTAGTGGAACCAAGGAAAAAAAAAGAGGTATCCTTGATTAAGG
AACACTGTTTAAACAGTGTGGTTTCCAAAACCCTGAAACTGCATTAGTGTAATAGAAG
ACTAGACACCTCGATACAAATAATGGTTACTCAATTCAAAACTGCCA

TDH2 terminator

TAAATTTAACTCCTTAAGTTACTTTAATGATTTAGTTTTTATTATTAATAATTCATGCTCAT
GACATCTCATATACACGTTTATAAAACTTAAATAGATTGAAAATGTATTAAAGATTCCTC
AGGGATTCGATTTTTTTGGAAGTTTTTGTTTTTTTTTCCTTGAGATGCTGTAGTATTTGG
GAACAATTATACAATCGAAAGATATATGCTTACATTCGACCGTTTTAGCCGTGATCATTAT
CCTATAGTAACATAACCTGAAGCATAACTGACACTACTATCATCAATACTTGTCACATGA
GAACTCTGTGAATAATTAGGCCACTGAAATTTGATGCCTGAAGGACCGGCATCACGGA
TTTTCGATAAAGCACTTAGTATCACACTAATTGGCTTTTCGC
