# Supplementary material for: Reconstruction of de novo verazine biosynthesis in Saccharomyces cerevisiae and computational analysis of cholesterol-binding geometry for 22R hydroxylation by VcCYP90B27 to dissect the cyclopamine biosynthetic pathway
Source: Eng Life Sci. 2026 Aug 12;26(8):e008. doi: 10.48130/els-0026-0008 (PMC13599646; doi:10.48130/els-0026-0008)
Supplement: Supplementary file 1 [file els-2026-26-8-0008-S1.zip › 10.48130_els-0026-0008-Suppl-Text1.pdf]

Text 1：Detailed methods of MD simulations:

To recreate the native microenvironment of the catalytic reaction and ensure the correct spatial positioning of the substrate and cofactor, the crystal structure of *Arabidopsis thaliana* sterol 22-hydroxylase (CYP90B1, PDB ID: 6A15), which shares high functional and structural homology, was selected as a reference template for multiple alignment and modeling. After screening for the VcCYP90B27 protein conformation with the optimal pLDDT confidence score, the molecular visualization softwares (UCSF ChimeraX and PyMOL) were used to perform full-backbone structural superposition of the AlphaFold-predicted model onto the 6A15 template. Subsequently, the atomic coordinates of the heme (HEM) cofactor and the substrate cholesterol from the template were transplanted in situ into the binding pocket of VcCYP90B27. Following local energy minimization to eliminate steric clashes, the initial VcCYP90B27-HEM-Cholesterol ternary reaction complex was successfully constructed.

An all-atom MD simulations were performed using the GROMACS software platform. The topology parameters of the receptor protein were described using the AMBER14SB all-atom, backbone dihedral-optimized force field. The small-molecule charges and topology parameters for the substrate cholesterol and the HEM cofactor were generated using the General AMBER Force Field , combined with partial charges calculated via the AM1-BCC method. The complex system was placed in a dodecahedral periodic water box with a minimum distance of 1.2 nm from the protein surface and was fully solvated using the TIP3P explicit water model. Subsequently, $Na^{+}$and Cl^{-} ions were randomly added to a concentration of 0.15 M to neutralize the total charge of the system and simulate a physiological saline environment. Before the

formal simulation, energy minimization was performed for a maximum of 50,000 steps using the steepest descent algorithm. Next, positional restraints were applied to the heavy atoms of the protein and ligand, followed by 100 ps of system equilibration in the NVT ensemble (constant volume, constant temperature of 300 K, using the V-rescale thermostat) and then in the NPT ensemble (constant pressure of 1.0 bar, constant temperature, using the Parrinello-Rahman barostat). After equilibration, all positional restraints were removed, and an unbiased production run of 1000 ns was carried out. The integration time step was set to 2 fs, and all covalent bond vibrations involving hydrogen atoms were constrained using the LINCS algorithm. Long-range electrostatic interactions were calculated using the Particle Mesh Ewald (PME) method, and the cutoff radius for short-range non-bonded van der Waals and electrostatic interactions was uniformly set to 1.2 nm. Simulation trajectories were recorded every 10 ps for subsequent analysis.

Using the trajectory analysis toolsuite included in GROMACS, time series of the RMSD of the protein backbone, the catalytic pocket binding site, and the substrate cholesterol (ligand) were extracted from the 1000 ns trajectory. The root-mean-square fluctuation (RMSF) of the full-length amino acid residues was also calculated to quantitatively assess the dynamic stability and local flexibility of the system in the aqueous environment. To investigate the stereoselective microscopic mechanism of the 22(*R*)-specific hydroxylation reaction, the gmx distance module was used to continuously and dynamically extract the three-dimensional Euclidean distances from the central heme iron (Fe) ion to the two prochiral hydrogen atoms (pro-*R* H and pro-*S* H) at the C22 site of the substrate from the entire MD trajectory. After smoothing the distance data, the kernel density estimation (KDE) method was used to calculate and plot the frequency density distribution curves.

The molecular mechanics/generalized Born surface area (MM-GBSA) method was employed to calculate the end-state binding free energy. Using the

gmx_MMPBSA script package, representative conformational snapshots were uniformly extracted from the stable segment of the MD trajectory where the simulation approached equilibrium for analysis. The total binding free energy (Δ Gbind) was decomposed into gas-phase van der Waals (vdW) energy, Coulomb electrostatic interaction energy, lipophilic energy (Lipo), and solvation free energy (Solv_GB). Furthermore, per-residue energy decomposition was performed to accurately identify hot-spot amino acids that provide key thermodynamic contributions to substrate anchoring. To further systematically deconstruct the restrictive network within the catalytic cavity, the frequencies of hydrophobic contacts and hydrogen bonds between pocket amino acids and the ligand were calculated based on MD trajectory contact data. A dynamic Sankey diagram was constructed using Python data analysis libraries.
