# Supplementary material for: Reconstruction of de novo verazine biosynthesis in Saccharomyces cerevisiae and computational analysis of cholesterol-binding geometry for 22R hydroxylation by VcCYP90B27 to dissect the cyclopamine biosynthetic pathway
Source: Eng Life Sci. 2026 Aug 12;26(8):e008. doi: 10.48130/els-0026-0008 (PMC13599646; doi:10.48130/els-0026-0008)
Supplement: Supplementary file 1 [file els-2026-26-8-0008-S1.zip › 10.48130_els-0026-0008-Suppl-FigureS1.pdf]

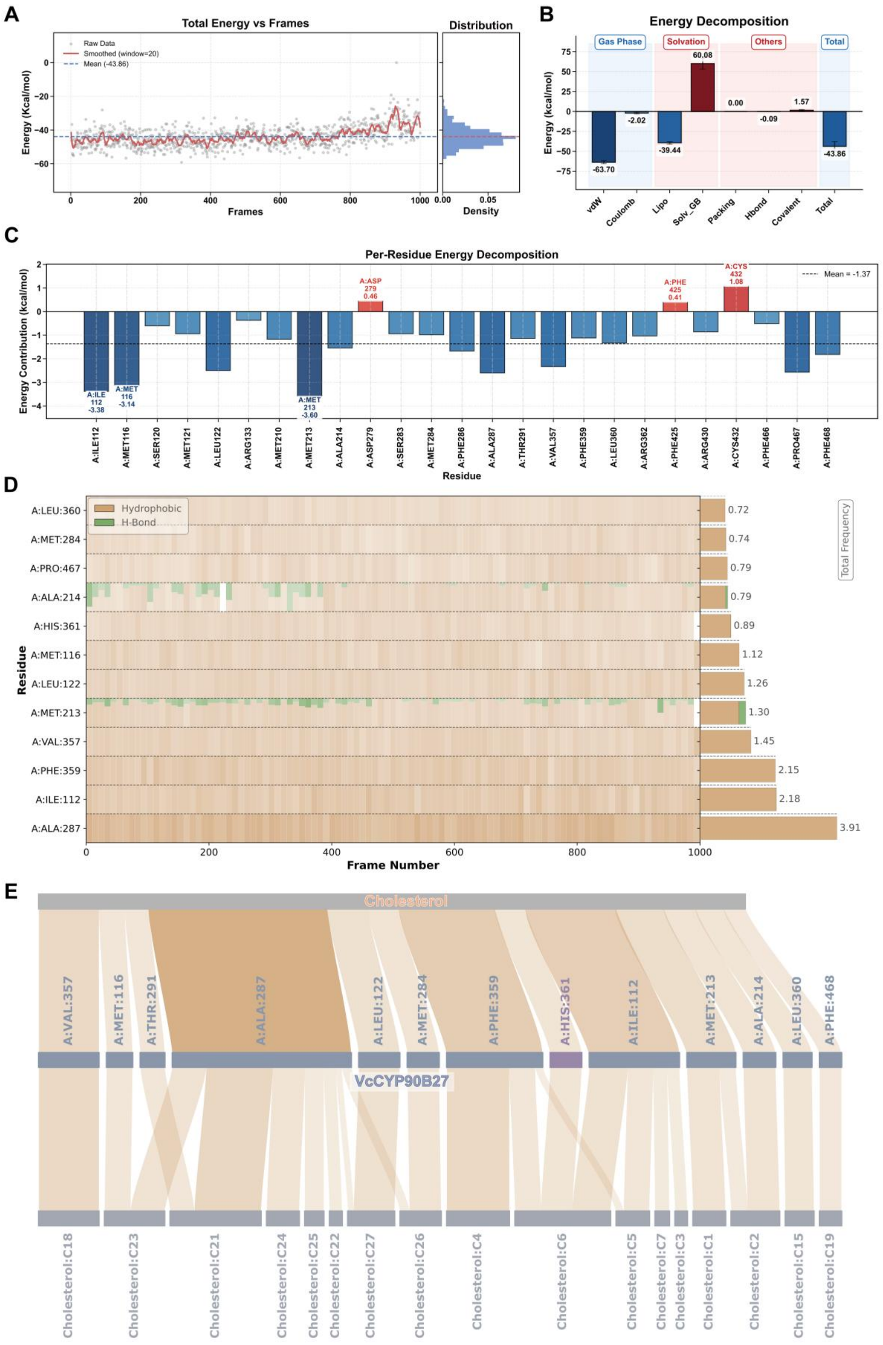

Figure S1 MM-GBSA binding-energy decomposition and non-covalent interaction network analysis.

(**A**) Time fluctuation series and probability distribution of the total binding free energy of the system throughout the 1000 ns MD trajectory. (**B**) Binding free energy calculated using the MM-GBSA method (total–43.58 kcal/mol) and its decomposition into physicochemical components. (**C**) Per-residue energy contribution bar chart of key amino acid residues to the total binding free energy. (**D**) Heatmap of non-covalent interaction frequencies between key amino acid residues in the binding pocket and the ligand. (**E**) A Sankey diagram mapping the dynamic interactions between specific carbon skeleton regions of the substrate and various protein residues.
